# Potential of carboxymethyl cellulose solution to increase the shale stability

**DOI:** 10.1038/s41598-023-44417-8

**Published:** 2023-10-13

**Authors:** AKM Badrul Alam, Yoshiaki Fujii, Nahid Hasan Dipu, Torin Chakma, Prodeepta Neogi, ASM Woobaid Ullah, Rini Asnida Abdullah

**Affiliations:** 1 Petroleum and Mining Engineering Department, Faculty of Civil Engineering, MIST, 9th Floor, General Mustafiz Tower, Mirpur Cantonment, Dhaka, 1216 Bangladesh; 2 Faculty of Engineering, Hokkaido University, Sapporo, Japan; 3 Geology Department, University of Dhaka, Dhaka, Bangladesh; 4 Faculty of Civil Engineering, Universiti Teknologi Malaysia, Skudai, Malaysia

**Keywords:** Civil engineering, Environmental impact

## Abstract

Slope failures in Bangladesh's Chittagong division are a growing concern, with fatalities increasing from 19 in 2000 to 162 in 2017 and projected to rise further. This study aims to identify the most vulnerable rock formation and assess Carboxymethyl Cellulose (CMC) as a solution for enhancing shale strength and mitigating slope failures. The research began by evaluating weathering susceptibility and stability of different rock layers, revealing the high instability of shale in the Bhuban Formation. Slake durability tests measured cation concentration to understand shale instability mechanisms. Laboratory experiments, including immersion tests and grained-and-molded shale specimens, examined CMC's potential to improve shale stability. Results indicated that the shale of the Bhuban Formation had the highest hammer value variations, indicating increased weathering susceptibility. Shale instability was attributed to illite layer dissolution, releasing K^+^. Intact shale specimens treated with CMC showed enhanced penetration resistance, shear strength, and deformation behavior, suggesting CMC's potential in increasing shale stability. Grained-and-molded shale specimens treated with CMC demonstrated increased shear strength, critical shear displacement, and contraction deformational behavior. Optical microscopy and scanning electron microscopy revealed the formation of cross-links between shale grains, contributing to improved shale stability. Further research is needed to explore the application of CMC for enhancing in situ rock slope stability. This study emphasizes the importance of addressing slope failures in the Chittagong division and provides insights into mitigating the risks through CMC-based interventions.

## Introduction

**Figure 1:**
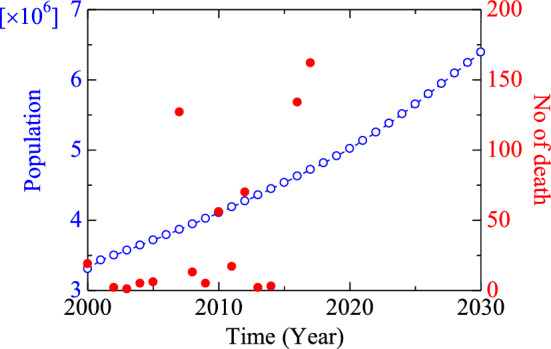
Population and number of death trends of the Chittagong Hill Tacks area, Bangladesh (Alam et al.^14^).

Slope failure in the Chittagong division of Bangladesh is a significant regional concern, with a steady increase in the number of fatalities from 19 in 2000 to 162 in 2017 (Fig. 1)^1,2^. This trend is expected to persist due to population growth and increased human activities in the area.

Slope failures in the region have been addressed from various perspectives, including GIS and remote sensing^3,4^, statistical and machine learning^5–11^, and soil mechanics^10,12,13^. However, the investigation of shale stability from a rock mechanics perspective of the region was initially conducted by our research group^14^.

Studies have investigated the borehole stability in shale^15–22^, hydration models and the influence of salinity on hydration^23,24^, factors affecting the mechanical properties of shale^25–32^, and failure criteria of shale^33,34^. Additionally, other studies have explored the impact of shale and other rock characteristics on underground openings^21,35–38^ in various regions. However, in the current works, the engineering geological approach has been used primarily to explore the shale's instability mechanism.

Carboxymethyl cellulose (CMC) has been utilized in the petroleum oil and natural gas industry for improving borehole stability through adjustments of drilling mud's physical properties under varying conditions^39–42^. Furthermore, CMC finds applications in civil construction^43–45^. However, the potential use of CMC for stabilizing shale slopes has not yet been established which is verified in present study.

## Methodology

**Figure 2:**
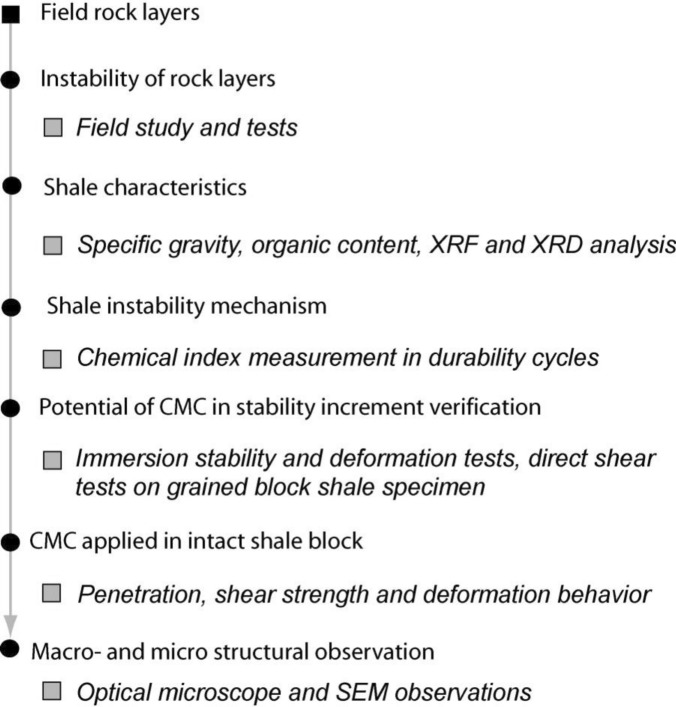
Methodology flowchart.

This study aims to comprehensively investigate the stability of shale rock layers and assess the potential of Carboxymethyl Cellulose (CMC) in enhancing shale stability to prevent slope failure. The research consists of several key steps (Fig. 2), beginning with field studies. Field tests were conducted to evaluate the instability and weathering susceptibility of shale, with particular emphasis on the Bhuban shale due to its high instability and susceptibility.

The shale samples underwent various analyses, including specific gravity measurements, assessment of organic content, XRF, and XRD analyses. Durability cycles were employed to investigate the mechanism of instability through chemical index measurements. The potential of CMC to enhance stability was evaluated through immersion stability tests, deformation tests, direct shear tests, and needle penetration tests on intact and grained-and-molded shale blocks. Macro- and micro-structural observations utilizing optical microscopy and SEM were conducted to gain insights into the mechanism of stability improvement. The details of specific methods, where necessary, are discussed in the respective sections.

## Instability of rock layers

The major rock layers in the Kaptai region of Chittagong were investigated and named based on the local areas, including the well-exposed section (Lichubagan), stream-cut section (Shilchari), and road-cut sections (Bergunia, Chandraghona, and Baraichari). Detailed field observations and Schmidt Rebound Hardness Tests were conducted on various rock layers to assess the relative variation in field strength and weathering effects.

**Figure 3:**
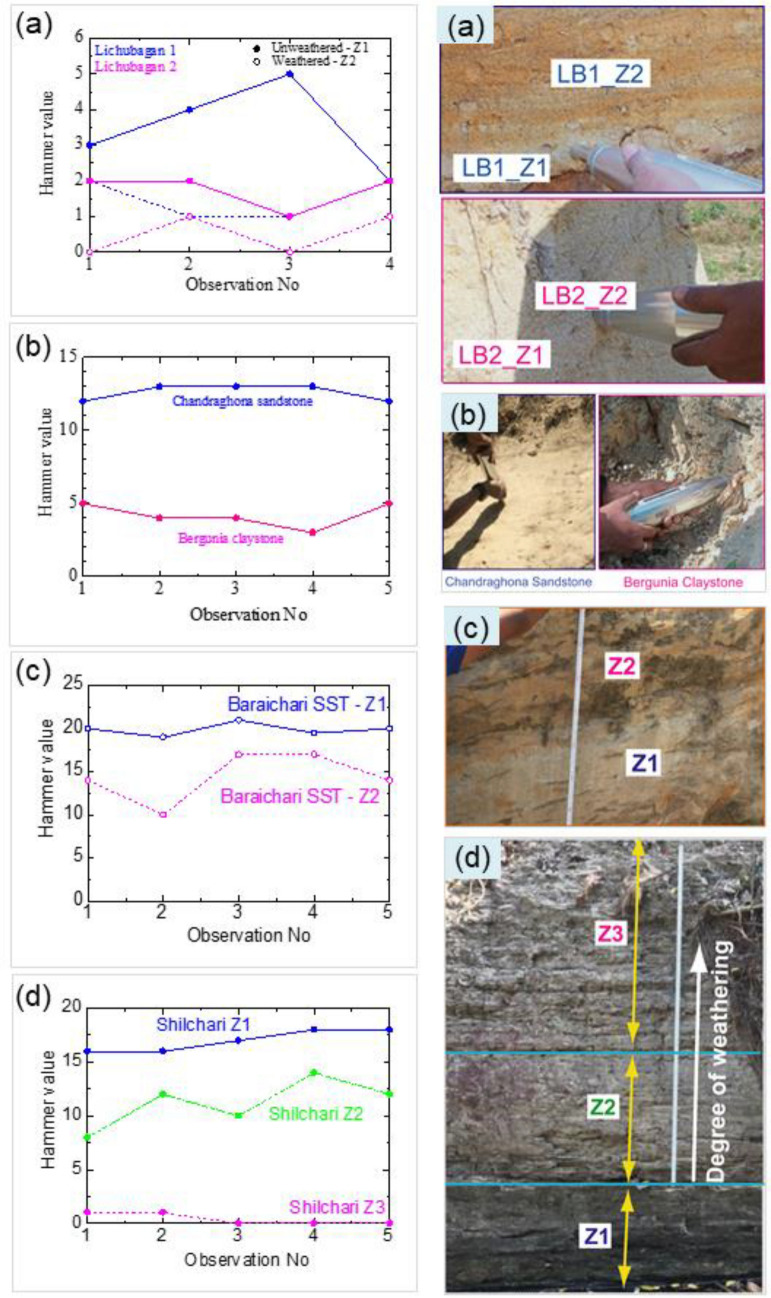
Rebound Hammer value (RH value) of Lichubagan compact (SST 1) and loose (SST 2) sandstone (**a**); Bergunia Claystone and Chandraghona Sandstone (**b**); Baraichari sandstone dominated rock layers (**c**); Shilchari shale dominated rock layers (**d**). *Z1* slightly weathered, *Z2* moderately weathered, *Z3* Weathered layers.

The sandstone at Lichubagan (LB) is classified into compacted (LB1) and loose (LB2) sandstones (Fig. 3). The rock layers are classified into less-weathered zones (Z1) and weathered zones (Z2) based on their grayish, yellowish, and reddish colors. The Schmidt Rebound Hardness (RH) values were higher in LB1 compared to LB2, and the values decreased in the weathered zones. The average decrease ranged from 3.5 to 1.5 in LB1 and from 1.8 to 0.5 in LB2.

The claystone at Bergunia (BG) is bluish-gray in color, while the brownish sandstone at Chandraghona (CG) exhibits a massive structure. The Schmidt Rebound Hardness (RH) value was more than twice as high in CG compared to BG (Fig. 3b).

**Figure 4:**
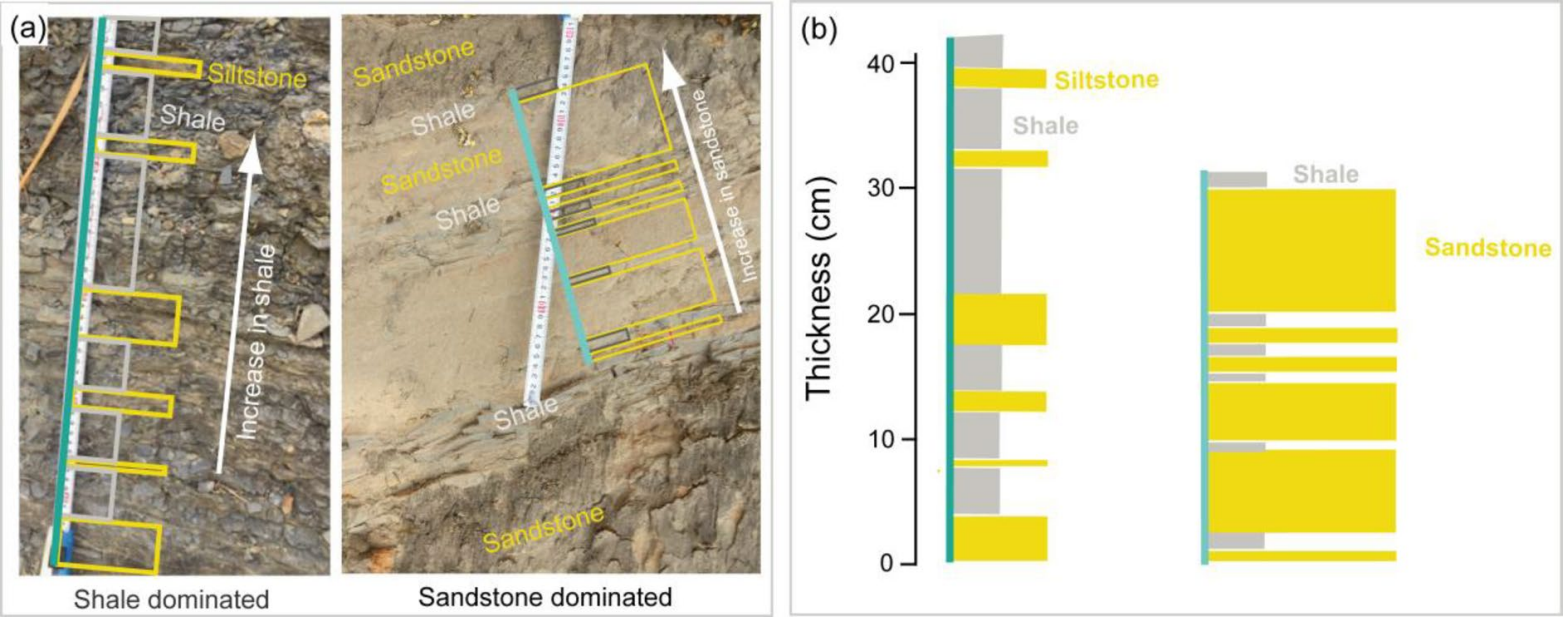
The shale (Shilchari) and sandstone (Baraichari) dominated rock layers in field exposure (**a**), comparison of litholog of the areas (**b**).

At Shilchori, grayish shale-dominated zones interlayered with light-yellowish siltstone were observed (Fig. 4). In contrast, at Baraichori, brownish and yellowish sandstone-dominated zones were interlayered with grayish shale (Fig. 4a). The shale-dominated rock unit is referred to as Shilchori–Shale–Siltstone (SC), while the sandstone-dominated rock unit is named Baraichori–Sandstone–Shale (BC) based on the lithology of the area (Fig. 4b). The BC layers were classified into less weathered zones (Z1) and weathered zones (Z2) based on the degree of weathering. Similarly, the layers of SC were classified as less weathered (Z1), moderately weathered (Z2), and highly weathered (Z3) zones. The average RH values were 19.9 for Z1 and 14.4 for Z2 in BC (Fig. 3c). For SC, the average RH values were 17 for Z1, 11.2 for Z2, and 0.4 for Z3 (Fig. 3d).

**Figure 5:**
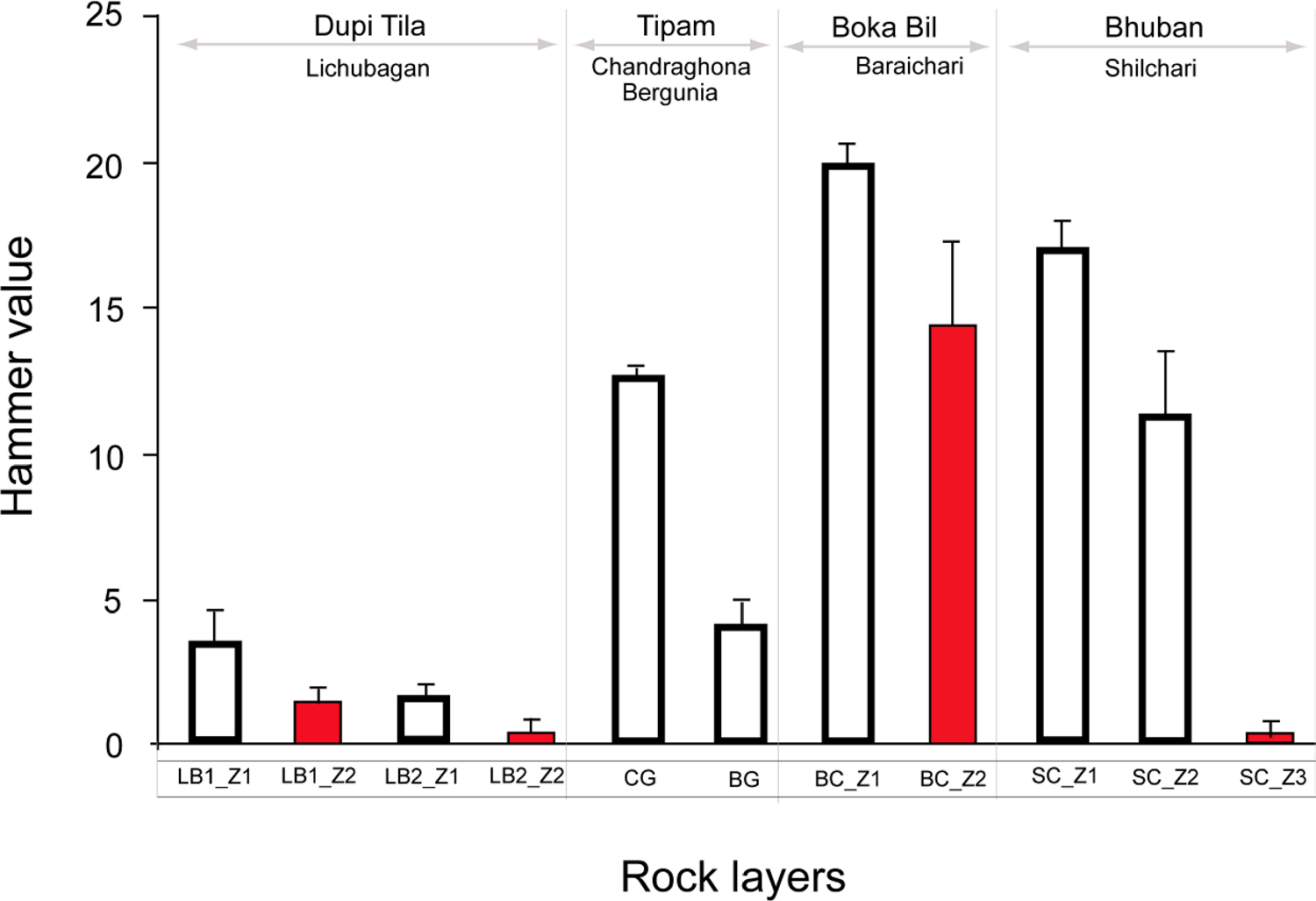
A comparison of the relative strength of the rock layers of the Kaptai, Chittagong, Bangladesh area. The red color indicates the weathered rock layer.

By correlating the local rock layers based on lithology with regional stratigraphy (Table 1), the sandstone at Lichubagan (LB) can be equivalent to the Dupi Tila formation, the claystone at Bergunia (BG) to the Girujan clay, the bluish-gray and brownish sandstone at Chandraghona (CG) to the Tipam sandstone. The sandstone-dominated rock unit, named Baraichori–Sandstone–Shale (BC), corresponds to the Boka Bil formation, while the shale-dominated rock unit, named Shilchori–Shale–Siltstone (SC), corresponds to the Bhuban formation. The RH values of these formations are shown in Fig. 5.

**Table 1:** Relative strength, stability, and weathering susceptibility of formations at Kaptai, Chittagong, Bangladesh.

SN	Study area		Regional stratigraphy		Relative strength (RH value)	Stability and susceptibility
	Local rock unit and location	Lithology	Formation	Lithology		
1	Lichubagan-sandstone (LB) *Lat 22° 30′ 31.39956"* *Long 92° 9′ 17.67132"*	Yellowish. reddish and brownish sandstone	Dupi Tila	Yellowish brown sandstone	3.5 ± 1.3 Z1; 1.5 ± 0.6 Z2, SST 1 1.8 ± 0.5 Z1; 0.5 ± 0.6 Z2, SST 2	Low stability
2	Bergunia-Claystone (BG) *Lat 25° 30′ 31.39956"* *Long 92° 9′ 15.87132"*	Bluish-gray massive claystone	Girujan Clay	Bluish gray claystone	4.2 ± 0.8 Z1	Moderately stable
3	Chandraghona-Sandstone (CG) *Lat 22° 29′ 27.14820"* *Long 92° 7′ 51.84696"*	Brownish compacted sandstone	Tipam sandstone	Yellowish brown sandstone	12.6 ± 0.5 Z1	Stable
4	Baraichari-Sandstone-Shale (BC) *Lat 22° 29′ 27.14820"* *Long 92° 7′ 51.750120"*	Sandstone-dominated rock layers and an alteration of shale	Boka Bil	Alteration of sandstone and shale with a calcareous band and minor siltstone	19.9 ± 0.7 Z1 14.4 ± 2.9 Z2	Stable and Less susceptible
5	Shilchari-Shale-Sandstone (SC) *Lat 22° 30′ 30.53484"* *Long 92° 9′ 16.36344"*	Grayish shale dominated rock layers and an alteration of siltstone	Bhuban	Alteration of light gray compacted shale and sandstone with siltstone	17.0 ± 1.0 Z1 11.2 ± 2.3 Z2 0.4 ± 0.5 Z3	Stable but Highly susceptible

Schmidt Rebound Hardness value (RH); compacted (SST 1) and loose (SST 2); Slightly weathered Z1, Weathered Z2, Z3.

The RH value of the Dupi Tila rocks indicates that the strength of the unweathered compacted sandstone was 2.3 times higher than that of the loose sandstone. In contrast, the strength of the Tipam sandstone at Chandraghona (CG) was three times higher than that of the Girujan clay at Bergunia (BG). The RH value was 1.4 times lower for the Boka Bil rocks at BG due to weathering. For the Bhuban rocks, the strength decreased by a factor of 1.5 due to moderate weathering, and in the case of highly weathered Bhuban rocks, the strength decreased by a factor of 43.

Based on the RH values and field observations, the estimated stability and weathering susceptibility of the Kaptai, Chittagong area can be concluded as follows: Dupi Tila has low stability, Tipam sandstone (CG) is stable, and Girujan clay (BG) is moderately stable. The Boka Bil formation is stable and has less weathering susceptibility. However, Bhuban shale is stable but highly susceptible to weathering. Therefore, the subsequent section and laboratory tests will focus on the characteristics of Bhuban shale and the potential of CMC to increase its stability.

## Shale characteristics and the instability mechanism by durability test

The Bhuban shale from the Bhuban Formation was characterized using various parameters. The specific gravity and organic content of the shale were determined using ASTM D854-14^46^ and ASTM D2974-14^47^, respectively. The grain size analysis was conducted following ASTM D 7928-17^48^, comparing the results with the Wentworth scale. Elemental composition was determined using XRF (Rigaku ZSX Primus), and the type of clay mineral was confirmed through XRD analysis (Ultima IV, Rigaku).

**Figure 6:**
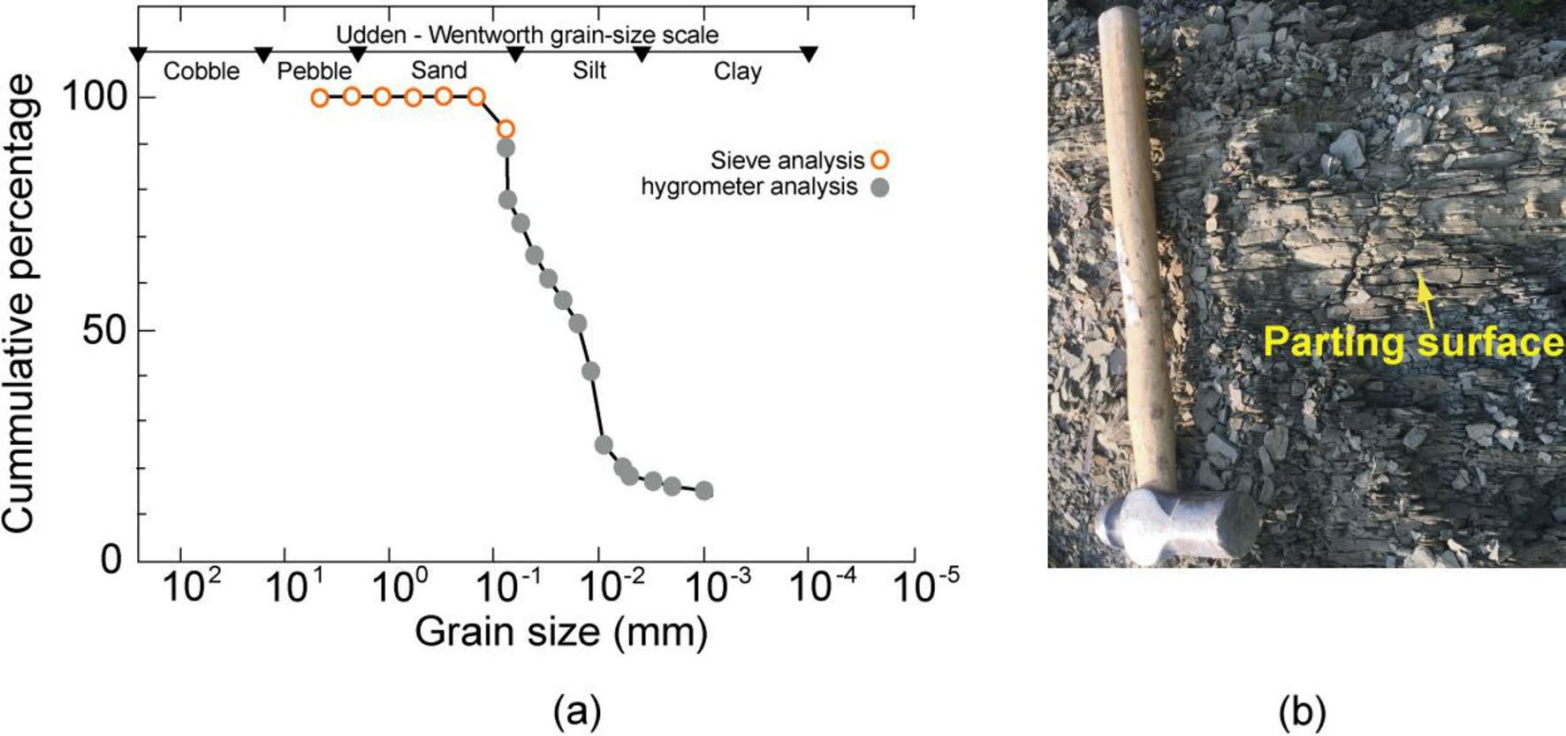
Grain size distribution and the shale in the field.

The specific gravity of the shale (*n* = 2) was found to be 2.63 ± 0.1, and the organic content was 3.6 ± 0.2%. The grain size distribution of the shale revealed that it mainly consisted of 76 wt% silt, 8 wt% fine sand, and 16 wt% clay (Fig. 6a). The presence of parting surfaces was observed in the shale (Fig. 6b), which is a common characteristic of shales.

**Figure 7:**
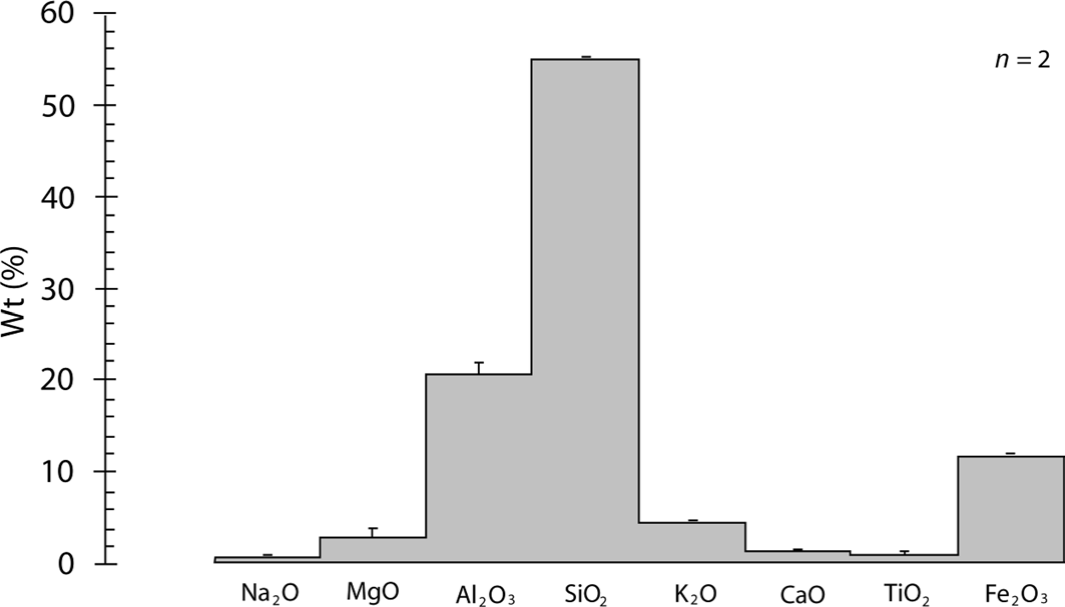
Elemental composition of the shale.

The major elements in the shale (*n* = 2) (Fig. 7) were found to be SiO_2_ (54.98 ± 1.02%), Al_2_O_3_ (20.85 ± 0.78%), Fe_2_O_3_ (12.01 ± 0.20%), K_2_O (4.60 ± 0.11%), MgO (3.14 ± 0.15%), with minor elements including CaO (1.42 ± 0.01%), TiO_2_ (1.11 ± 0.13%), Na_2_O (0.78 ± 0.08%), and others (1.13 ± 0.01%). The higher percentage of K_2_O in the shale is noteworthy.

**Figure 8:**
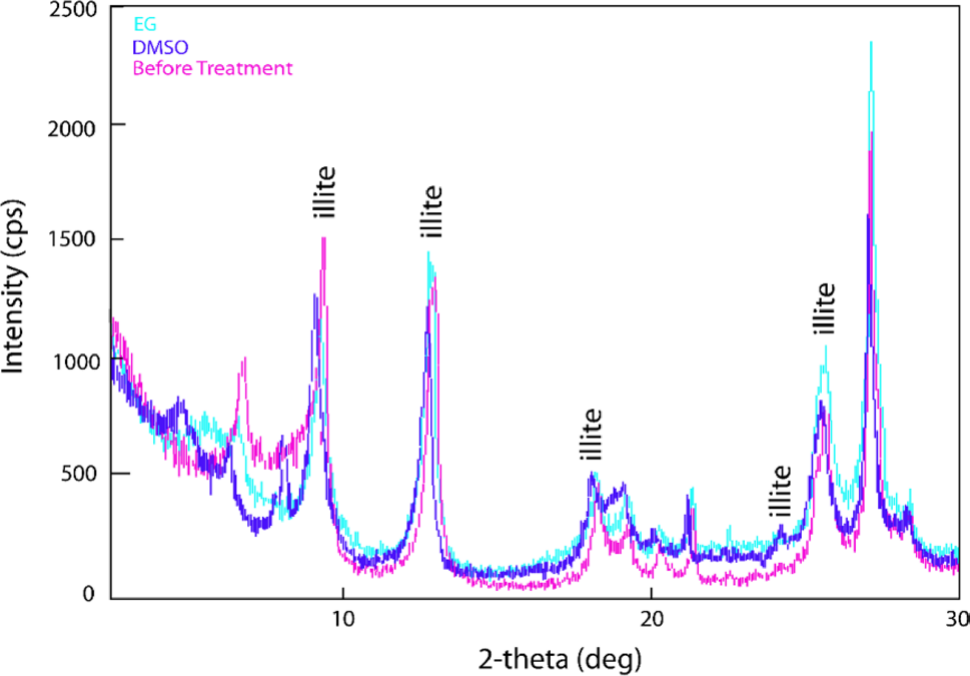
Illite Peaks in treated (*EG* ethylene glycol, *DMSO* di-methyl-sulpho-oxide) and non-treated shale.

**Figure 9:**
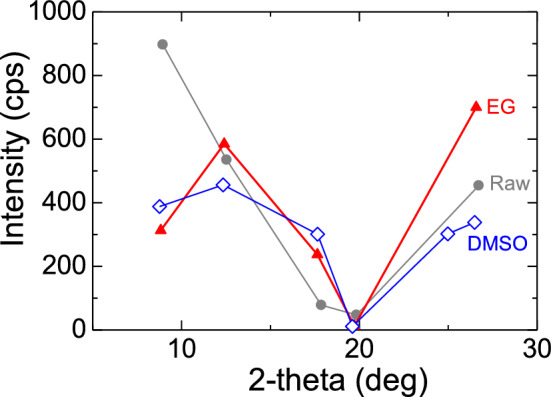
The intensity in Treated and non-treated shale.

The XRD spectra confirmed the presence of illite peaks (Fig. 8) following the guidelines provided by the US Geological Survey^49^. The intensity and position of the illite peaks increased slightly in ethylene glycol (EG), and a new peak appeared in dimethyl sulfoxide (DMSO) (Fig. 9). The changes in peak intensity and position after treatment with EG and DMSO indicate the presence of illite within the 10–30 2-theta (deg) range, which is consistent with the higher K_2_O content observed in the XRF analysis (Fig. 7).

To investigate the instability mechanism of the shale, durability tests were conducted using chemical index measurements. Ten samples (approximately 5 × 3 × 2 cm^3^ in size) were used for each set of durability test (total two sets), were placed in a stainless-steel mesh horizontal drum that was partially immersed in water. The drum was rotated according to the standard method described in ISRM^50^. The Durability Index (DI) was calculated as the Eq. (1). Various parameters were measured, including temperature, pH, resistivity, total dissolved solids (TDS), and concentrations of Na^+^ and K^+^. The concentrations were determined using an atomic absorption spectrophotometer (AA-6800, Shimadzu). The ambient temperature was maintained at 31 ± 1 °C, and the solid–liquid ratio was approximately 1:5.5 g/ml.

1 $$DI=\frac{{M}_{af}}{{M}_{bf}}\times 100$$

where *DI* is durability index; *M*_af_ is dry weight of samples after the durability test; *M*_bf_ is the dry weight of samples before the durability test.

**Figure 10:**
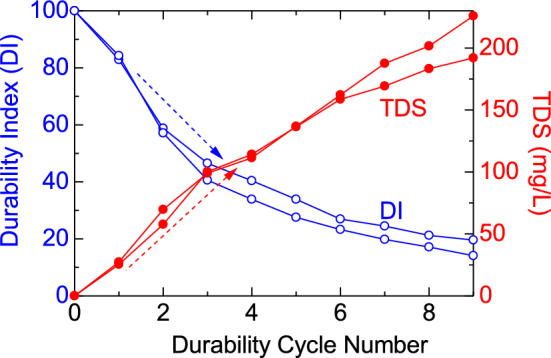
Total dissolved solid (TDS) in durability cycles.

**Figure 11:**
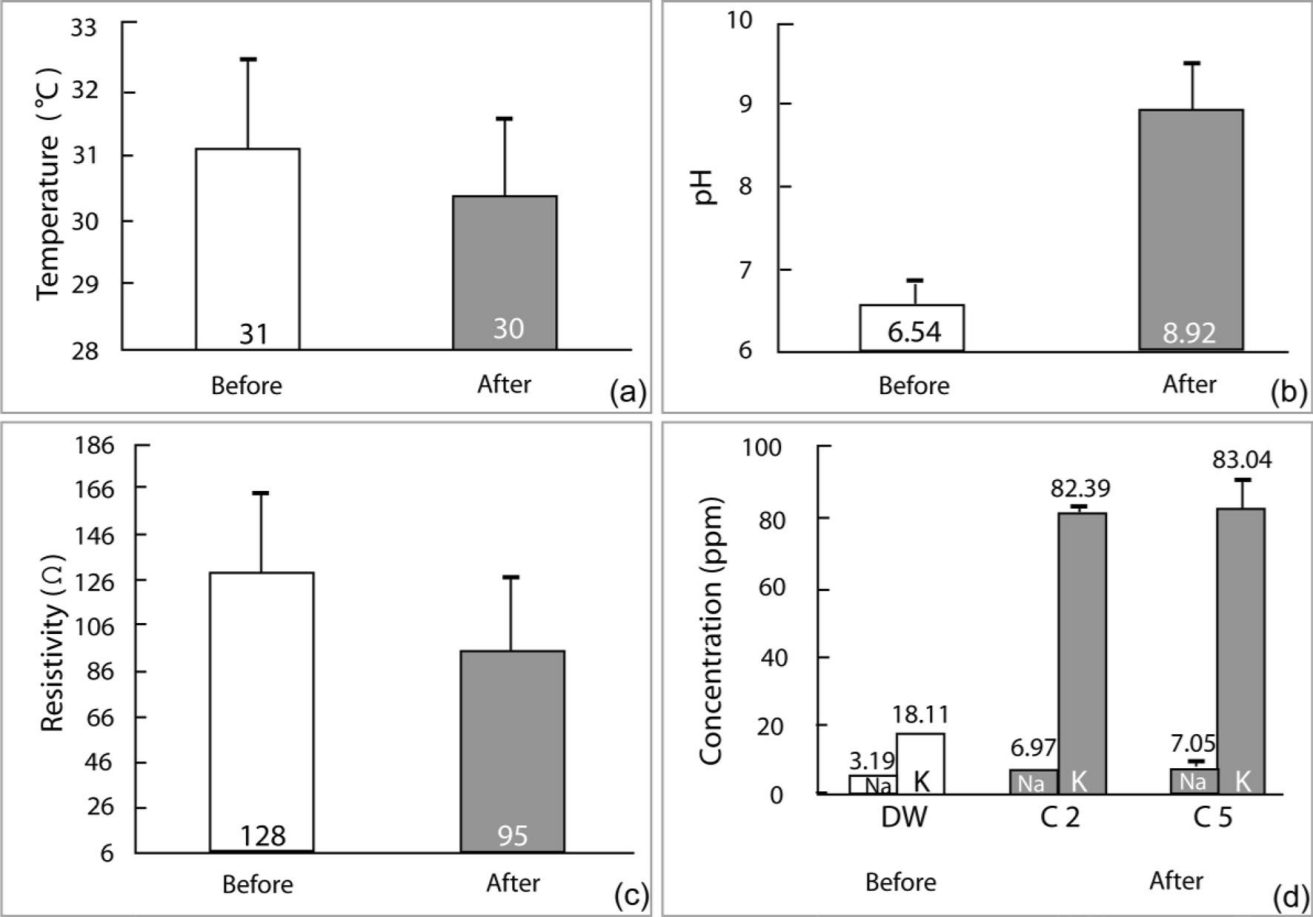
Change in temperature, pH, and resistivity before and after durability cycles and Na^+^ and K^+^ concentration in durability cycles.

The DI of the shale showed a decreasing trend that correlated with the durability cycles, as depicted in Fig. 11. Furthermore, the content of Total Dissolved Solids (TDS) exhibited a significant increase as the durability cycles advanced, as shown in Fig. 10. The temperature decreased during the test (Fig. 11a). The pH increased and resistivity decreased (Fig. 11b,c). The concentrations of Na^+^ and K^+^ showed an increase, with the latter increasing more significantly than the former (Fig. 11d) which is also reflected in resistivity decrease. The dissolution of K^+^ from the illite layer might have contributed to the disintegration of the rock into smaller pieces, leading to a higher concentration of K^+^ after slaking, as K^+^ is interlayered within the illite^51^.

## Potential of CMC in increasing stability of shale

To assess the potential of CMC in increasing shale stability, grained-and-molded shale blocks were prepared to minimize structural and compositional inhomogeneity. Immersion tests, deformation characteristics, and direct shear tests were conducted.

The shale was ground using a ball mill (Universal Ball Mill UB32, Yamato) and sieved through a #100 mesh. The resulting ground shale powder was then mixed with CMC solution (Poly) or distilled water (No-poly) at a ratio of 1 g of shale powder to 0.416 ml of CMC solution or water. The mixture was poured into molds and dried at 105 °C for one day.

### Immersion stability test

Poly and No-poly shale specimens measuring 3 × 3 × 3 cm^3^ were placed in an acrylic box. The box was filled with distilled water for up to 360 min to observe their stability. The room temperature was maintained at 25 °C. The concentrations of Na^+^ and K^+^ in the water were measured.

**Figure 12:**
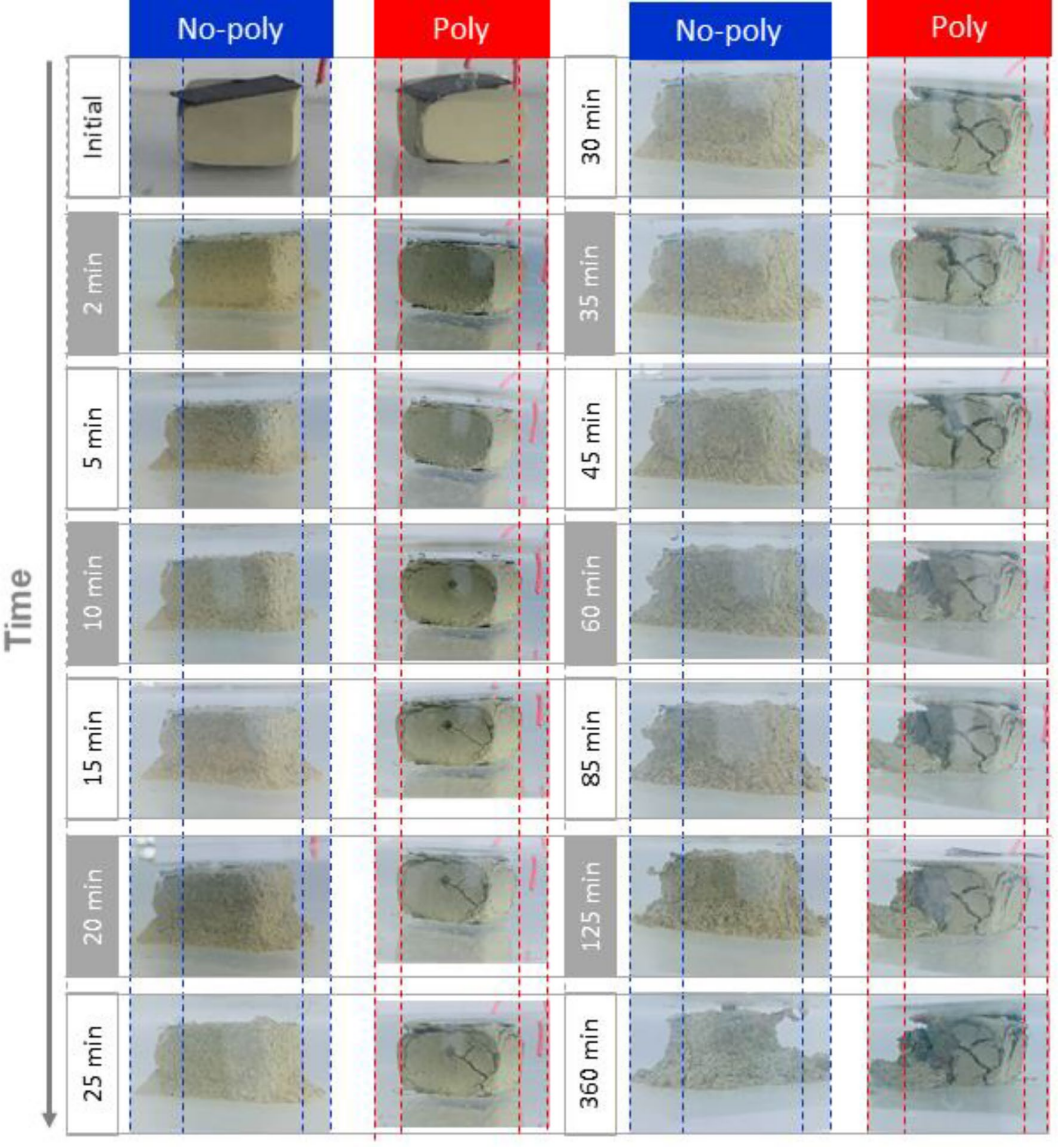
Stability of water-immersed No-poly and Poly specimens.

The observations are depicted in Fig. 12. For the Poly specimen, visible deformation nucleation occurred within 10 min. Subsequently, a fracture appeared in the deformation nucleus within 15 min. The number of fractures increased to two within 25 min and four within 30 min. A portion of the fractured block disintegrated at 60 min but remained stable for up to 360 min. On the other hand, for the No-poly specimen, grain disintegration occurred immediately and continued throughout the entire 360-min period.

**Figure 13:**
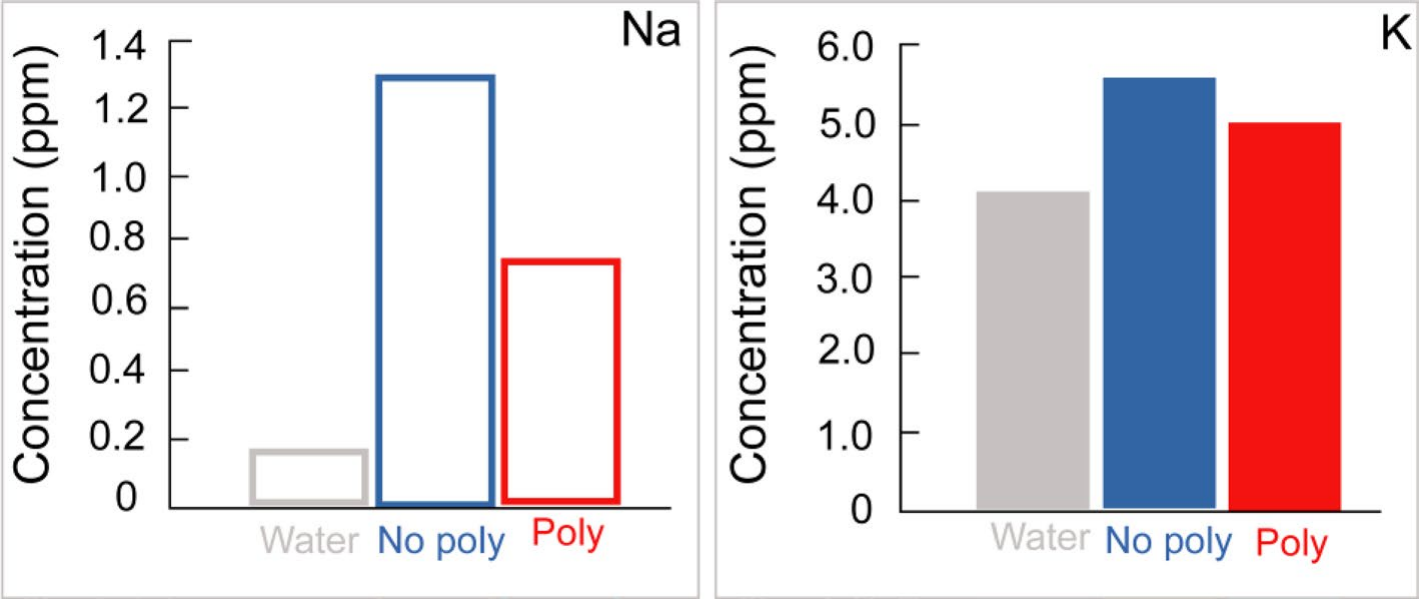
Na^+^ and K^+^ concentration of the immersed water for No-poly and Poly specimens.

The concentrations of Na^+^ and K^+^ were significantly higher for the No-poly specimen compared to the Poly specimen (Fig. 13). The dissolution of these cations may be the cause of fracturing in water.

### Immersion deformation test

Specimens measuring 6 × 6 × 1.5 cm^3^ were used. The top surface of the specimens was left open, while the sides were sealed with silica sealant. A strain gauge (KEG-10-120-D16-11N5C2, EDX-200A, KYOWA) was attached to the top surface of each specimen. The specimens were then immersed in water at atmospheric pressure, and the deformation was observed at a constant room temperature of 25 °C.

**Figure 14:**
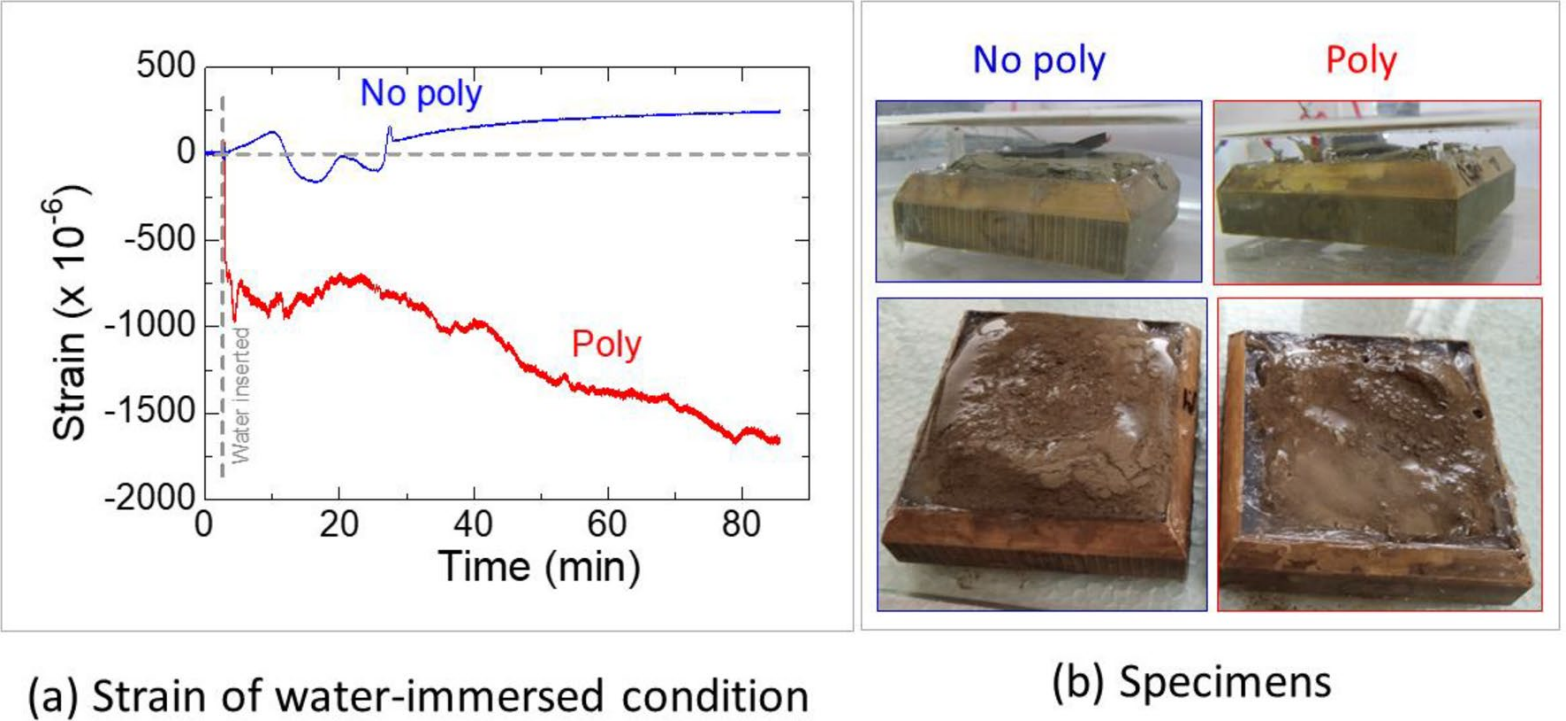
Deformation of No-poly and Poly specimens at top-surface.

The No-poly specimen showed slight expansion during the immersion test, while the Poly specimen exhibited contraction (Fig. 14a). The maximum vertical expansion observed in the No-poly specimen was 5 mm, whereas the Poly specimen only exhibited a maximum expansion of 1 mm (Fig. 14b). The clay minerals in shale were the main reason of the expansion and CMC bonding in clay and the grains might have prevent the expansion rather increased in contraction.

### Direct shear tests

Blocks measuring 6 × 6 × 1.5 cm^3^ were subjected to consolidation for 24 h at normal stresses of 25, 50, or 100 kPa. The consolidation was performed either in distilled water (wet conditions) or in air (dry conditions) to achieve the target stress state. Subsequently, a direct shear test was conducted on the consolidated specimens at a strain rate of 0.045 mm/min using a loading frame (ELE, EL26-2114). The tests were conducted at a room temperature of 25 °C.

**Figure 15:**
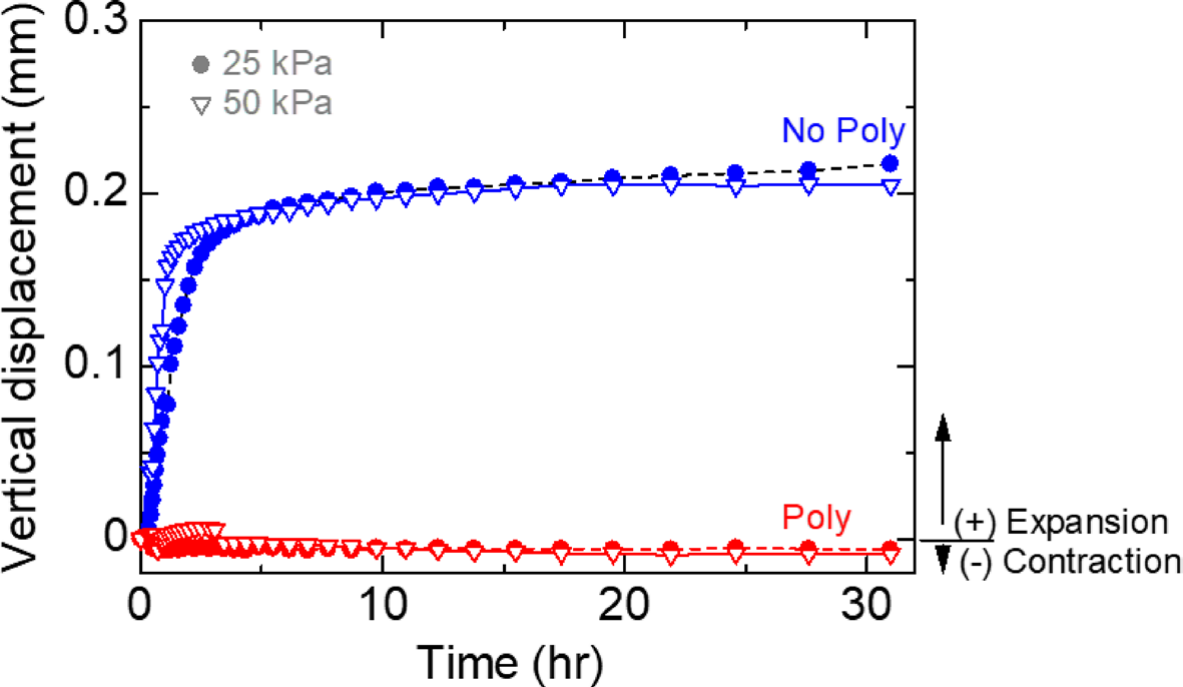
The vertical displacement of water-immersed No-poly and Poly specimens under normal stress.

During the consolidation process, only the vertical displacement allowed under constant normal stress was continuously monitored. Under wet conditions, the No-poly specimens exhibited significant expansion, while the Poly specimens showed slight contraction (Fig. 15).

**Figure 16:**
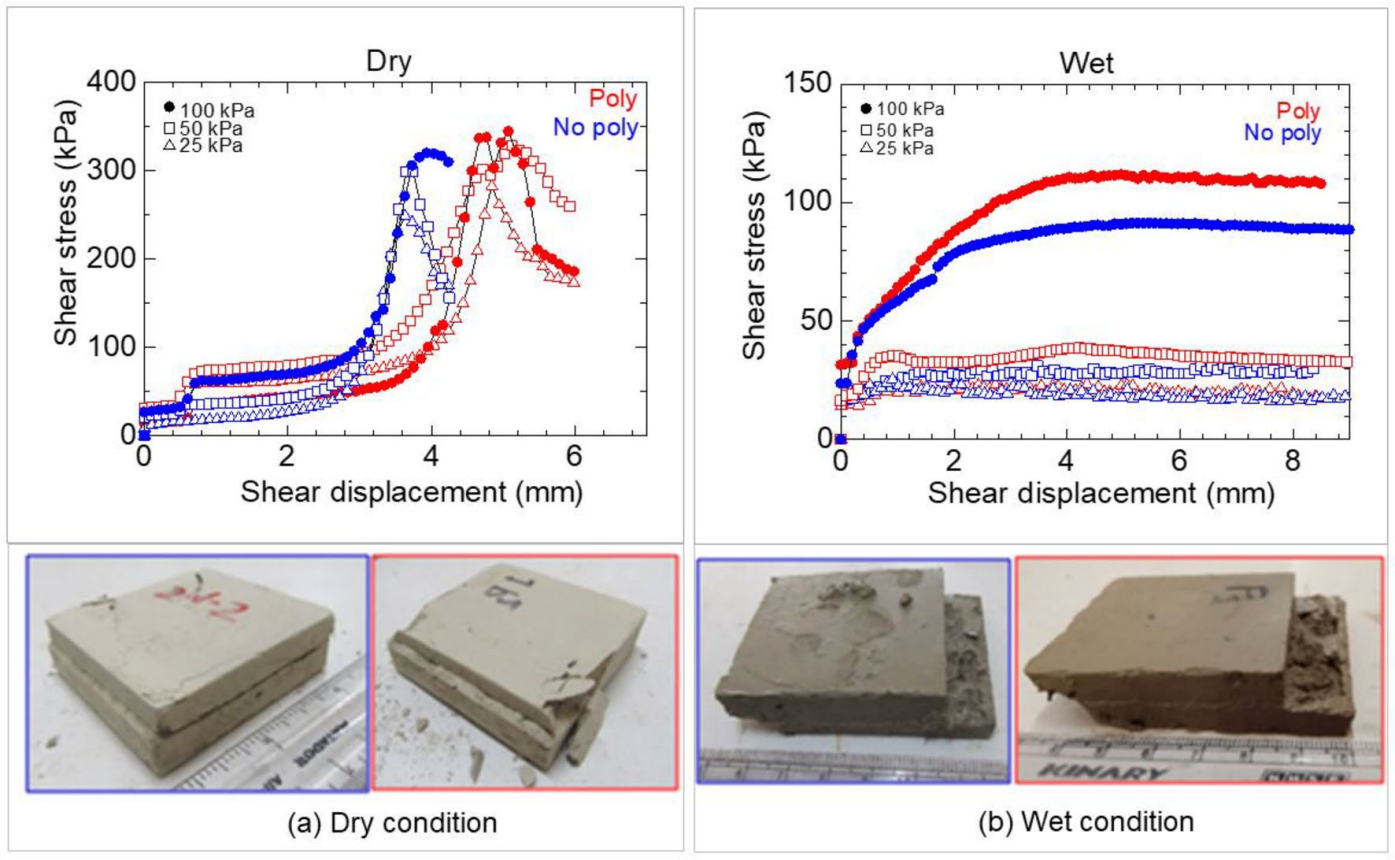
Shear strength of No-poly and Poly specimens in dry (**a**) and wet conditions (**b**).

**Figure 17:**
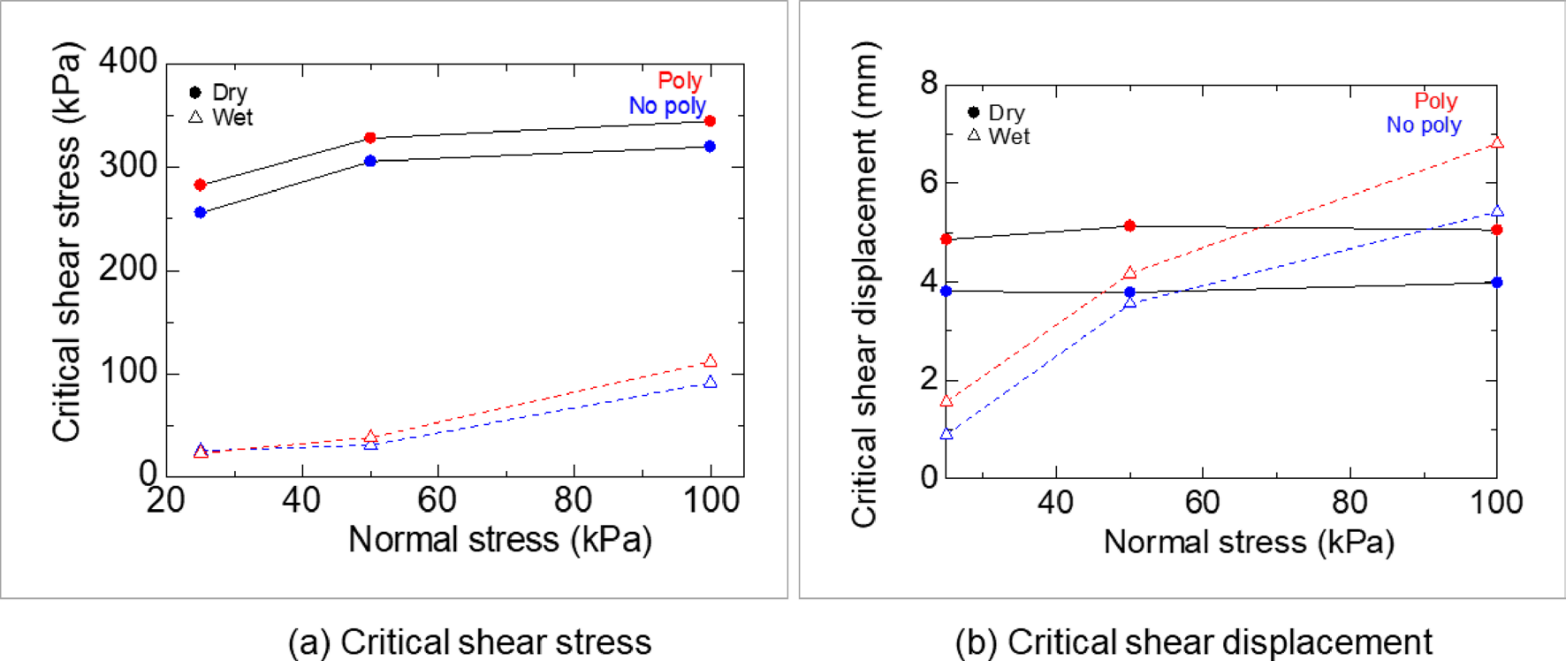
Critical shear stress (**a**) and displacement (**b**) of No-poly and Poly specimens in direct shear tests.

Under dry conditions, the shale specimens displayed strain-softening behavior, as evidenced by the observed stress drop (Fig. 16a) and the presence of distinct shear planes in the specimens after the test for both the Poly and No-poly specimens (Fig. 16b). The strength (Fig. 17a) and critical displacement (Fig. 17b) of the Poly specimens were higher compared to those of the No-poly specimens.

Under wet conditions, the shale exhibited ductile behavior (Fig. 16b). The strengths of the No-poly and Poly specimens were initially similar under the lowest normal stress condition. However, the strengths of the Poly specimens gradually increased compared to those of the No-poly specimens as the normal stress increased (Fig. 16a). The critical displacement of the Poly specimens was also greater than that of the No-poly specimens.

## Application to shale block

### Immersion and needle penetration tests

Approximately 5 × 3 × 4 cm^3^ intact shale blocks were dried at 105 °C for 24 h and then immersed in distilled water or a 6 mg/L CMC solution for 14 days to assess their stability. Needle penetration tests were performed using 81-B0102/B (Controls).

**Figure 18:**
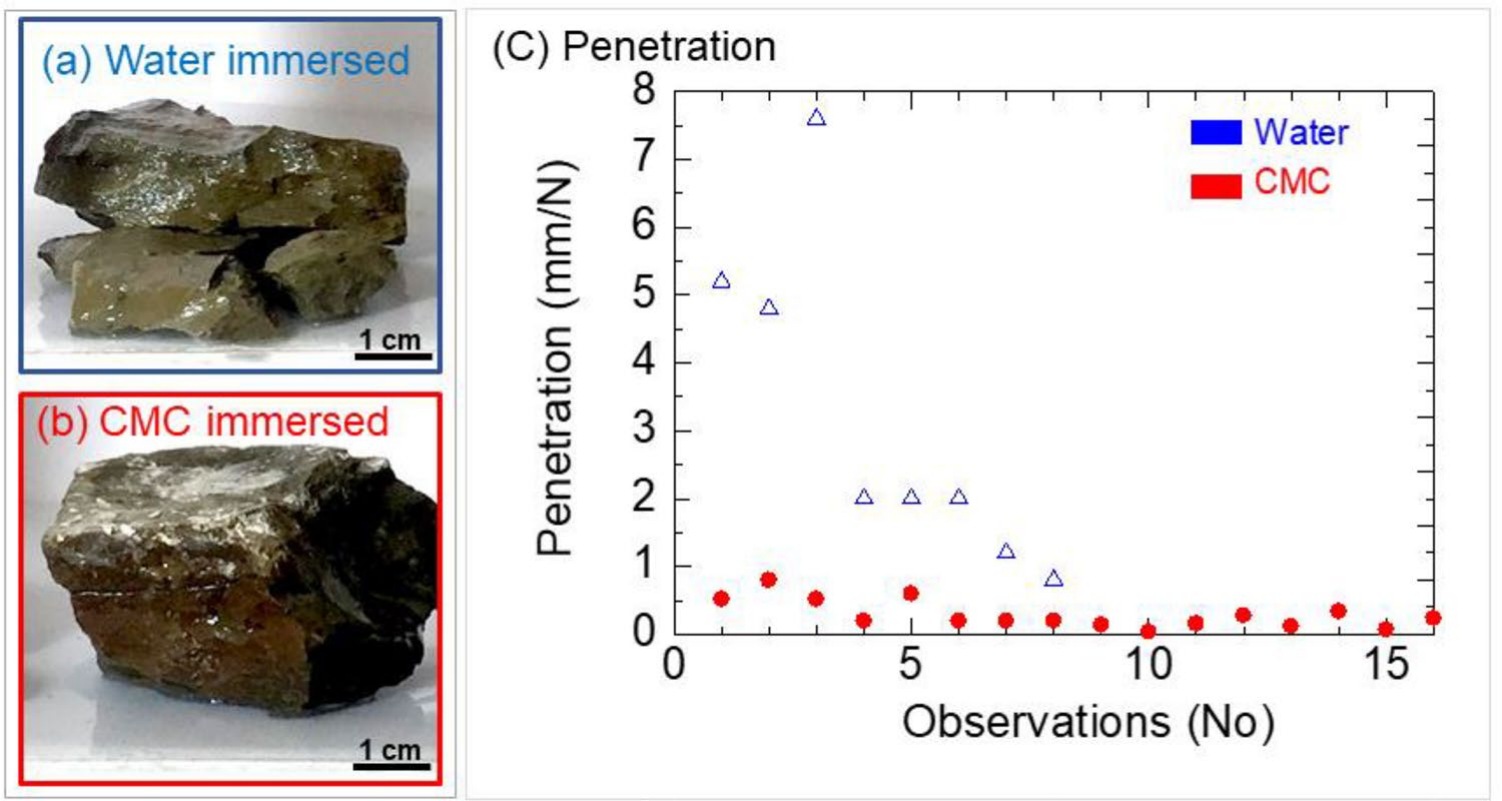
Shale blocks after immersion in distilled water (**a**) and CMC solution (**b**), and the penetration (**c**).

Immersion of the shale block in water resulted in its disintegration (as shown in Fig. 18a), and it exhibited a high needle penetration value of 3.20 ± 2.39 mm/N (as shown in Fig. 18c). In contrast, when the shale block was immersed in CMC, it remained intact (Fig. 18b), and the needle penetration value was significantly reduced to 0.29 ± 0.21 mm/N.

The difference in needle penetration measurements between CMC and water was substantial, with the penetration in CMC being 11 times lower than that in water. These findings suggest that CMC may serve as an effective stabilizing agent for shale blocks.

### Immersion deformation test

A 65 × 45 × 35 cm^3^ shale block was placed in a shear box, and water or a 6 mg/L CMC solution was poured and maintained for several tens of hours until the displacement transducer output stabilized, indicating saturation at a normal stress of 25 kPa.

**Figure 19:**
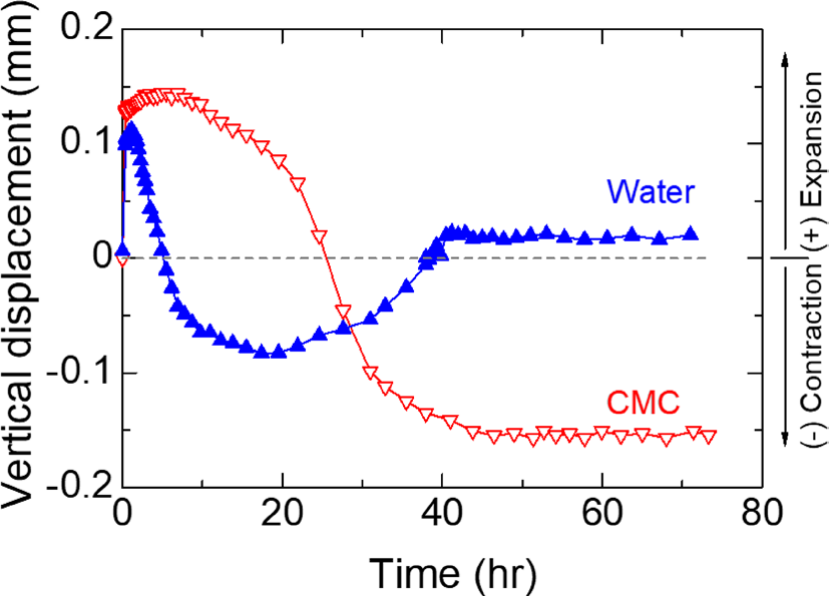
Saturation characteristics of block shale in water or CMC solution immersed at consolidation stage.

In water, the shale block specimen initially expanded by 0.11 mm within the first 1.1 h of immersion (Fig. 19). This was followed by a contraction phase, reaching a maximum contraction of − 0.08 mm after 17.4 h of immersion. However, the specimen underwent another expansion phase and eventually stabilized at a value of 0.02 mm after 40.5 h of immersion. The behavior of the specimen in water is characterized by a complex pattern of initial expansion, contraction, and subsequent expansion.

On the other hand, the shale block specimen exhibited higher initial expansion (0.14 mm within the first 5.5 h of immersion) in the CMC solution. It then underwent a continuous contraction phase until reaching a maximum contraction of − 0.15 mm after 43.8 h of immersion. Unlike in water, the specimen in the CMC solution did not undergo any subsequent expansion after the contraction phase.

The expansion of the specimen in water following the contraction phase may be indicative of instability.

### Direct shear test

**Figure 20:**
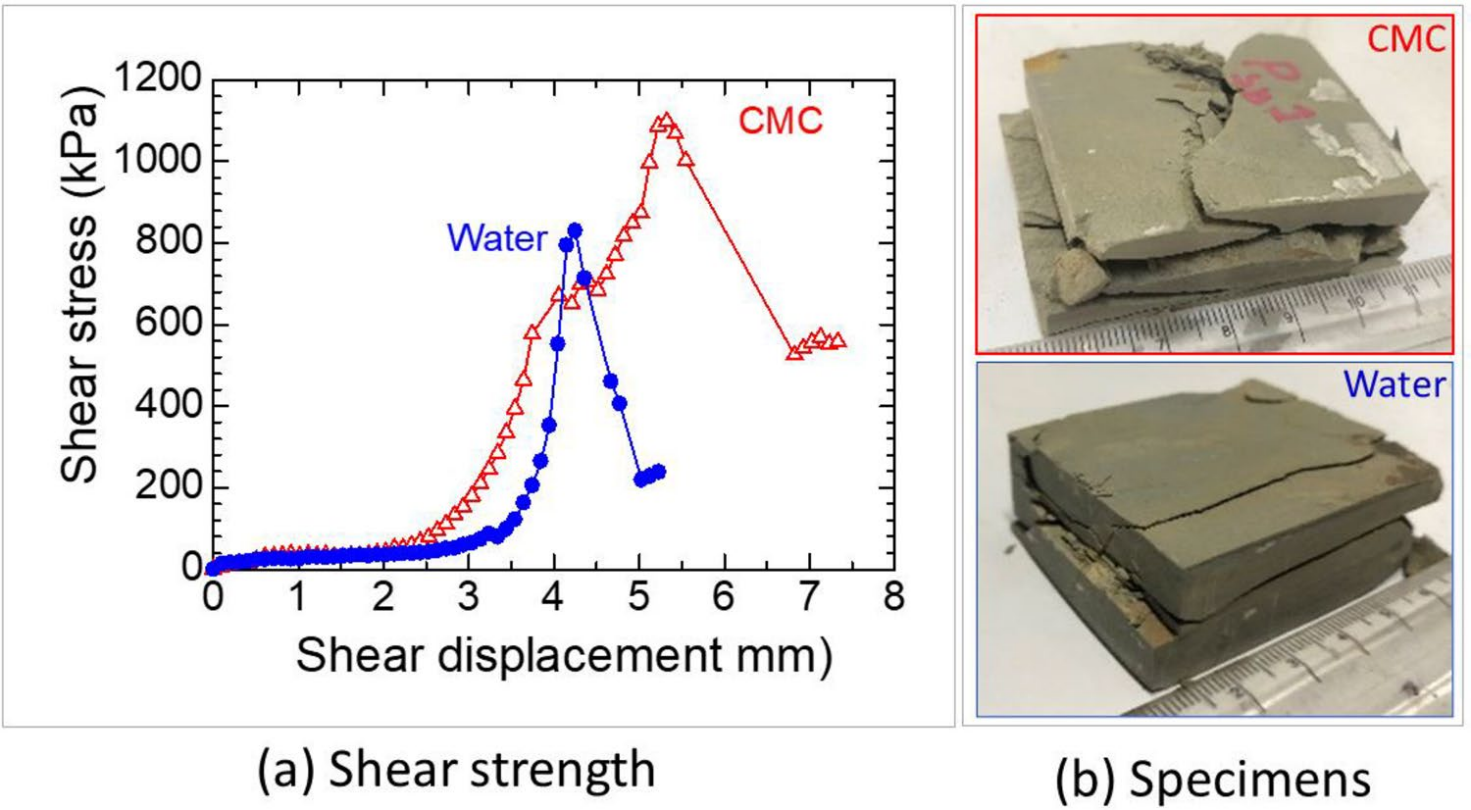
Strength and critical shear displacement increase due to polymer.

Specimens measuring 65 × 45 × 35 cm^3^ were dried at 105 °C for 24 h, and subsequently, a direct shear test (Fig. 20) was conducted at a strain rate of 0.0455 mm/min under two different conditions: immersion in water and immersion in a 6 mg/L CMC solution.

In the water-immersed scenario, the shale block displayed a shear strength of 830 kPa at a displacement of 4.2 mm (refer to Fig. 20). Contrastingly, when subjected to the CMC-immersed scenario, the shale block demonstrated a notably higher shear strength of 1098 kPa at a larger displacement of 5.3 mm. This highlights a substantial enhancement in shear strength through the utilization of the CMC solution.

## Microstructure

In the first step, the CMC solution was poured onto two shale grains placed on a glass slide. The grains were then observed using an optical microscope (LEICA, DM750P) under both cross-polarized and plane-polarized light after 24 h. The morphology of the Poly and No-poly specimens was also examined using a SEM with a Hitachi 2600SN microscope.

**Figure 21:**
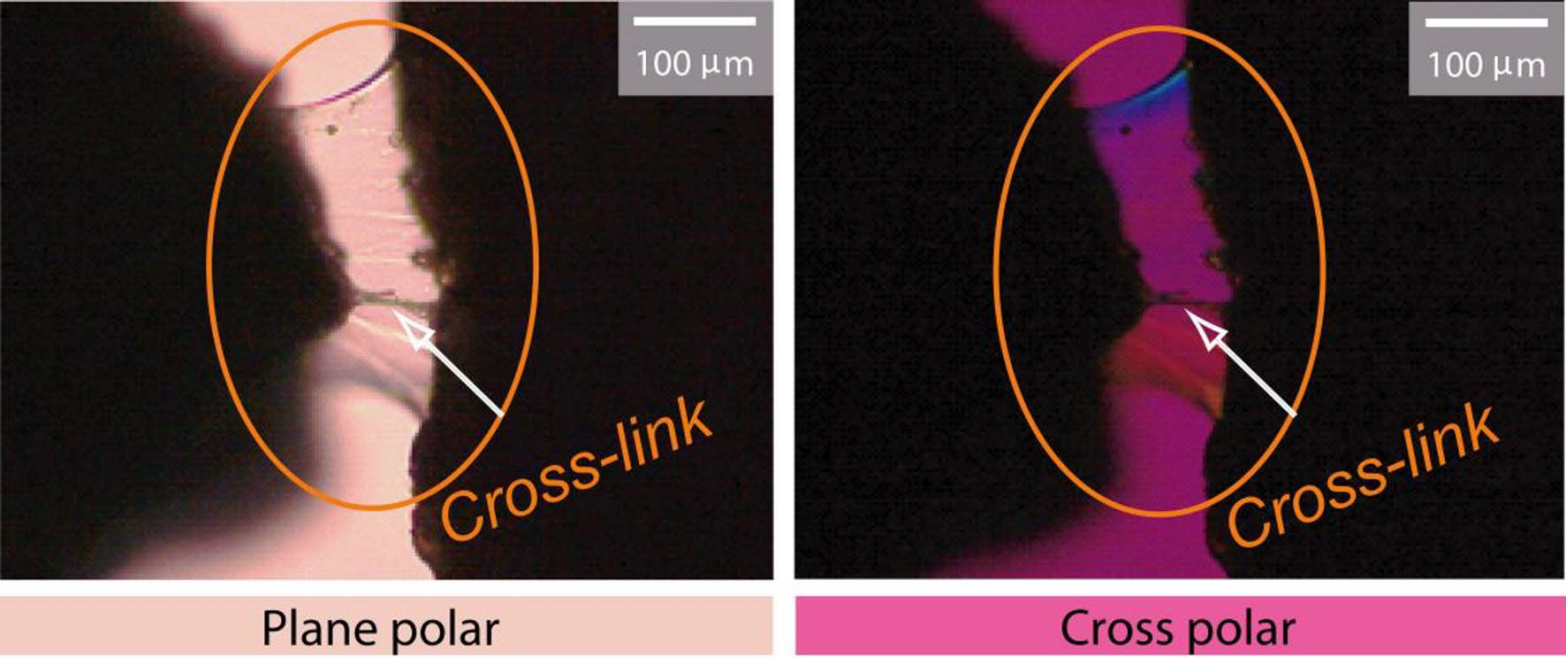
Cross-links in shale grains after applying CMC solution which was absent beforehand under the optical microscope.

**Figure 22:**
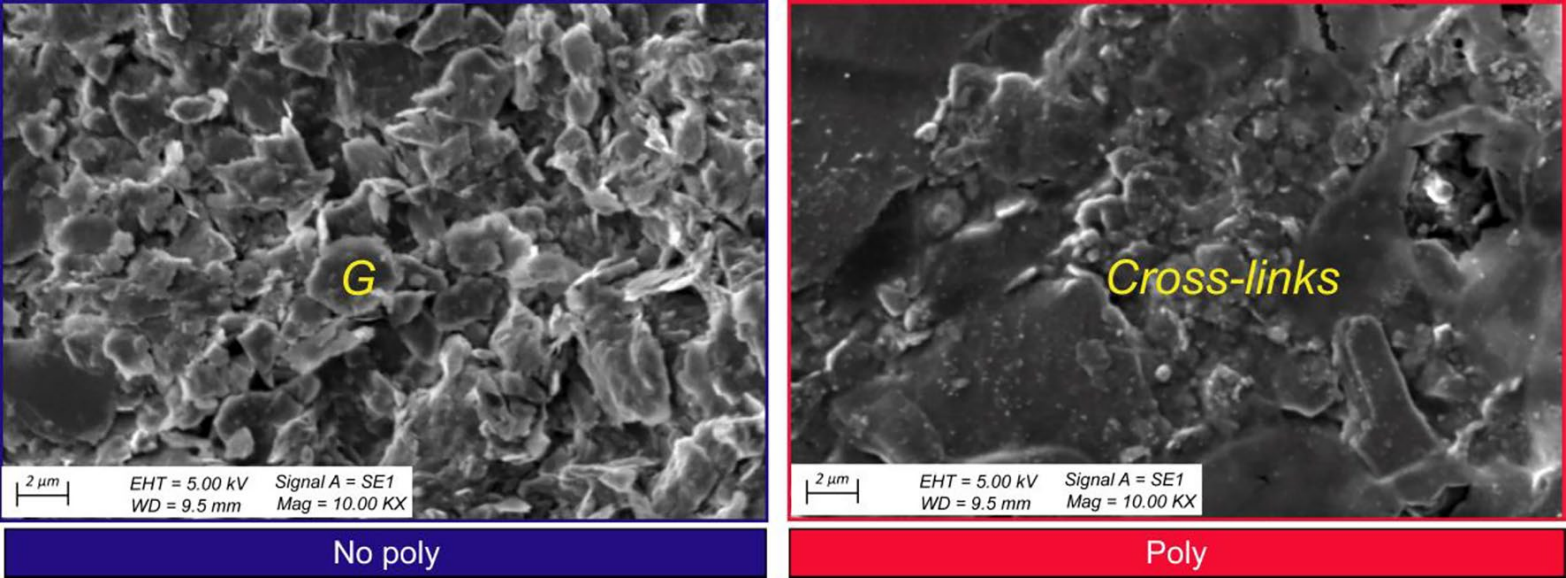
Cross-links in SEM micrograph. *G* Individual grain.

The observations of the shale grains with CMC solution under the optical microscope revealed the presence of cross-linking bonds between the shale grains, which were induced by the CMC (Fig. 21). SEM observations of the grained-and-molded shale specimens, both the No-poly and Poly variants, showed distinct differences. The No-poly specimen exhibited shale flakes, while the Poly specimen displayed bonding between the flakes, indicating the presence of cross-links (Fig. 22). These results strongly suggest that CMC acts as an effective bonding agent for shale, enhancing its stability.

## Discussion

Shale is a complex sedimentary rock that contains various minerals, including clay minerals, quartz, feldspar, calcite, and pyrite. The composition of shale can vary depending on factors such as its depositional environment, tectonic setting, and diagenetic history. In the case of the Bhuban shale, located in the study area, it was found to have a clay content of 16% and an organic matter content ranging from 3.4 to 3.8%. The dominant mineral identified in the shale was illite, which is supported by the higher percentage of K_2_O obtained from the XRF analysis (Fig. 7) and peaks in XRD analysis (Figs. 8, 9). The Bhuban shale was characterized as a silty shale with low organic matter content but a significant presence of illite.

When considering the stability of shale formations during drilling operations, the water absorption characteristics play a crucial role. Specific water absorption can lead to shale instability, as explained by Sun et al.^52^. The mechanism of hydration swelling and its influence on crack propagation have been investigated in studies on Lower Silurian Longmaxi Formation (LF) shale by Liu et al.^53^. In the case of the Bhuban shale, the instability mechanism is attributed to the dissolution of K^+^ ions from the illite interlayers, which occurs as a result of water absorption. This mechanism is supported by the observed increase in K^+^ concentration (Fig. 11) after the durability test.

Understanding the composition and behavior of shale formations, such as the Bhuban shale, is crucial for assessing their stability. The presence of clay minerals, particularly illite, and the potential for water absorption-induced instability highlight the importance of appropriate stabilization measures. The experiments conducted in this study, including durability tests, immersion tests, deformation tests, and direct shear tests, provide valuable insights into the effectiveness of CMC solution as a stabilizing agent for shale. The observed cross-linking bonds between shale grains (Figs. 21, 22) and the enhanced shear strength exhibited by the CMC-immersed shale specimens (Fig. 16) support its potential as a beneficial additive for improving shale stability.

The use of Carboxymethyl Cellulose (CMC) in drilling fluids has been widely studied in the petroleum industry, including its effects on wellbore stability. Park et al.^54^ conducted research on the impact of CMC on wellbore stability and found that it significantly reduced the risk of shale hydration and associated instability. This suggests that incorporating CMC into drilling fluids can contribute to improved drilling performance and reduced fluid loss rates, thereby enhancing wellbore stability. Furthermore, the environmental implications of CMC-based drilling fluids have been investigated. Sadiq et al.^55^ conducted a study to evaluate the toxicity and environmental impact of CMC and found that its use did not cause significant harm to the environment or exhibit notable toxicity.

In the present study, the focus was on exploring the use of CMC in shale slope stability. Various experiments were conducted to investigate its effects on stability, shear strength, and critical shear displacement. The grained-and-molded shale specimens are uniform and exhibit consistent shapes. Therefore, we anticipate obtaining accurate results regarding the potential of CMC to enhance shale strength. Nevertheless, natural shale, in its unaltered state, possesses varying permeability characteristics and should be subjected to testing as well, even if the specimens may exhibit non-uniformity and irregular shapes. The results indicate that the incorporation of CMC can enhance shale stability.

In intact shale, the use of CMC resulted in reduced needle penetration (Fig. 18), improved deformation behavior (Fig. 19), increased shear strength, and critical shear displacement (Fig. 20). These findings were further supported by the contraction deformational behaviors (Figs. 14, 15), increased stability (Fig. 12), enhanced shear displacement, and critical displacement (Figs. 16, 17) observed in the grained-and-molded shale specimens.

The formation of cross-links between shale grains, as observed through optical microscopy and SEM (Figs. 21, 22), is believed to be the primary reason for the increased shale stability when CMC is used. These cross-links contribute to improved bonding between shale particles, enhancing the overall stability of the shale formations.

Overall, the findings from this study align with previous research and support the potential benefits of incorporating CMC in shale slope stability, including improved stability, shear strength, and critical shear displacement. Given that the cost of CMC is approximately $1.5 (USD) per kilogram and, in this study, we found that the 6 mg/L CMC solution is beneficial, it may be cost-effective considering the short as well as long term effect of a slope failure in shale.

**Figure 23:**
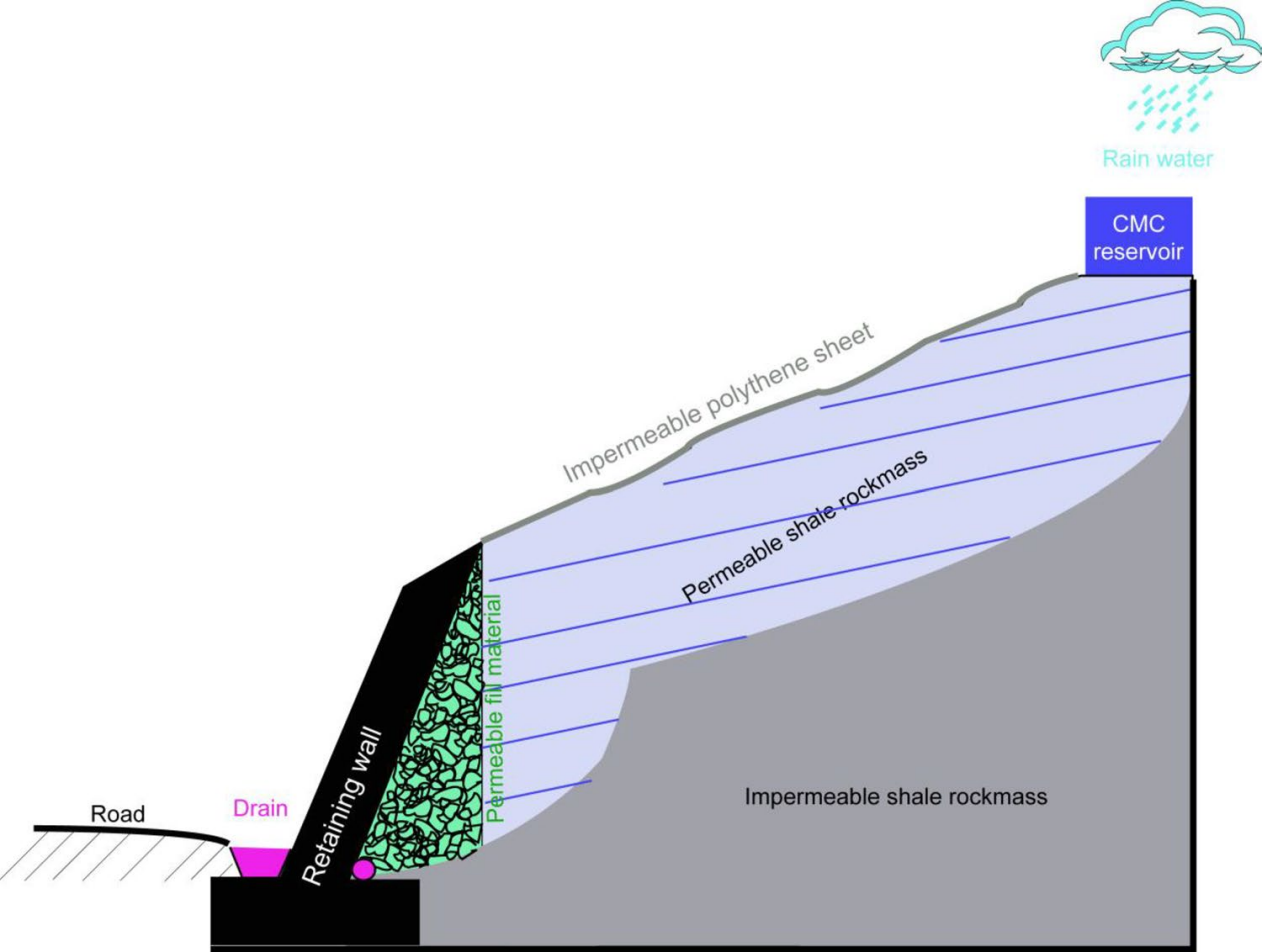
Conceptual (schematic) diagram of CMC solution filed application.

The schematic representation depicted in Fig. 23 illustrates an innovative approach to utilizing a CMC solution in practical applications. In this method, a retaining wall is strategically combined with a CMC reservoir which is positioned at the summit of a slope that is covered with impermeable polythene sheeting. The primary objective of this design is to facilitate the infiltration of the CMC solution into the porous regions of a shale rock mass. The incorporation of a retaining wall not only serves as a structural support localized to the slope but also plays a crucial role in enabling the deliberate infiltration process. By optimizing the interaction between the CMC solution and the shale rock mass, this approach contributes to enhancing the overall regional stability of the shale slope. This innovative technique likely holds significance in soft rock engineering for controlling slope stability. But, in the future, the conceptual design could be used in a case study for a more in-depth examination or comprehension.

## Conclusion

In conclusion, this study investigated the potential use of carboxymethyl cellulose (CMC) to enhance the stability of shale and prevent slope failure in the study area. The key findings of the study are summarized as follows:

1. The shale from the Bhuban Formation exhibited higher susceptibility to weathering, as indicated by higher variations in relative humidity (RH). This suggests that the shale is prone to instability.
2. Slaking of the shale resulted in the production of alkali metal cations, as evidenced by an increase in total dissolved solids (TDS), pH, and concentrations of Na^+^ and K^+^. The dissolution of K^+^ from the illite layer is believed to be a major factor contributing to shale instability.
3. The stability, shear strength, and critical shear displacement of grained-and-molded shale specimens mixed with CMC solution showed contraction and increased stability. This supports the effectiveness of CMC in enhancing shale stability.
4. Experimental results on intact shale demonstrated that the incorporation of CMC led to improvements in needle penetration resistance, shear strength, and deformation behavior. These findings further suggest that CMC has the potential to increase the stability of shale.
5. Optical microscopy and scanning electron microscopy (SEM) observations revealed the formation of cross-links between shale grains when CMC was present. These cross-links are believed to be responsible for the increased shale stability.

Based on these findings, it is recommended to further investigate the use of CMC to enhance the stability of in situ rock slopes. The optimal concentration is not being considered in this study and will be studied in next study. Continued research in this area could provide valuable insights into the application of CMC as a stabilizing agent for shale rockmass.

## Data Availability

The datasets used and/or analyzed during the current study are available from the corresponding author on reasonable request.
